# Supplementary material for: Vacuoles provide the source membrane for TORC1-containing signaling endosomes
Source: J Cell Biol. 2025 Mar 7;224(5):e202407021. doi: 10.1083/jcb.202407021 (PMC11893502; doi:10.1083/jcb.202407021)
Supplement: Table S1 — shows yeast strains used in this study. [file jcb_202407021_tables1.docx]

**Table S1. Yeast strains used in this study**

| **ID** | **Genotype** | **Source** |  |
| --- | --- | --- | --- |
| YL516 | [BY4741/2] MATa; *his3∆1 leu2∆0 ura3∆0* | (Binda et al., 2009) |  |
| yRL795 | [YL516] *mNeonGreen-TOR1* | This study |  |
| MP1632 | [YL516] *tor1∆::kanMX* | (Hatakeyama et al., 2019) |  |
| yRL897 | [yRL795] *atg18∆::kanMX* | This study |  |
| RKH94 | [YL516] *GFP-GTR1* | (Hatakeyama et al., 2019) |  |
| yRL392 | [RKH94] *atg18∆::kanMX* | This study |  |
| RKH158 | [YL516] *PIB2^200^-EGFP* | (Hatakeyama et al., 2019) |  |
| yRL399 | [RKH158] *atg18∆::kanMX* | This study |  |
| RKH486 | [YL516] *his3∆1::GFP-SCH9^1-183^::SpHIS5* | (Chen et al., 2021) |  |
| yRL402 | [RKH486] *atg18∆::kanMX* | This study |  |
| yRL803 | [YL516] *IVY1-mNeonGreen::HIS3* | This study |  |
| yRL1230 | [yRL803] *atg18∆::kanMX* | This study |  |
| yRL1173 | [YL516] *VPS4-mNeonGreen::hphNT1* | This study |  |
| yRL1233 | [yRL1173] *atg18∆::kanMX* | This study |  |
| yRL1170 | [YL516] *URA3::P_NOP1_-GFP-VPS21* | This study |  |
| yRL1201 | [yRL1170] *atg18∆::kanMX* | This study |  |
| yRL159 | [YL516] *SEC7-mCherry::natNT2* | This study |  |
| yRL1372 | [yRL159] *atg18∆::kanMX* | This study |  |
| yRL1380 | [yRL795] *natNT2::P_NOP1_-mCherry-ATG18* | This study |  |
| yRL1058 | [yRL795] *atg21∆::kanMX* | This study |  |
| yRL1060 | [yRL795] *hsv2∆::kanMX* | This study |  |
| yRL1053 | [yRL795] *atg2∆::kanMX* | This study |  |
| yRL1055 | [yRL795] *atg9∆::kanMX* | This study |  |
| yRL1063 | [yRL795] *vps26∆::kanMX* | This study |  |
| yRL1066 | [yRL795] *vps29∆::kanMX* | This study |  |
| yRL1069 | [yRL795] *vps35∆::kanMX* | This study |  |
| yRL1383 | [yRL795] *VPS26-mCherry-V5::kanMX* | This study |  |
| yRL913 | [yRL795] *ura3∆0::P_RNR2_-tetR-NLS-tup1-P_7tet.1_-tetR-NLS-URA3 P_ATG18_::hphNT1-P_7tet.1_-3xFLAG-ATG18* | This study |  |
|  |  |  |  |
| yRL1190 | [yRL795] *ura3∆0::P_RNR2_-tetR-NLS-tup1-P_7tet.1_-tetR-NLS-URA3 3FLAG-ATG18* | This study |  |
|  |  |  |  |
| yRL910 | [yRL795] *ura3∆0::P_RNR2_-tetR-NLS-tup1-P_7tet.1_-tetR-NLS-URA3 P_ATG18_::hphNT1-P_7tet.1_-ATG18* | This study |  |
|  |  |  |  |
| yRL1153 | [yRL910] *VPH1-mCherry-V5::kanMX* | This study |  |
| yRL1192 | [yRL910] *natNT2::P_TEF2_-mCherry-VPS21* | This study |  |
| yRL1194 | [yRL795] *natNT2::P_NOP1_-mCherry-ATG22* | This study |  |
| yRL1197 | [yRL910] *natNT2::P_NOP1_-mCherry-ATG22* | This study |  |
| yRL1270 | [yRL910] *natNT2::P_GAL1_-mCherry-ATG22* | This study |  |
| yRL1287 | [BY4741] MATa; *his3∆1 leu2∆0 met15∆0 mNeonGreen-TOR1* *ura3∆0::P_RNR2_-tetR-NLS-tup1-P_7tet.1_-tetR-NLS-URA3 P_ATG18_::hphNT1-P_7tet.1_-ATG18* | This study |  |
|  |  |  |  |
|  |  |  |  |
| yRL1299 | [BY4741] MATa; *his3∆1 leu2∆0 met15∆0 mNeonGreen-TOR1 ura3∆0::P_RNR2_-tetR-NLS-tup1-P_7tet.1_-tetR-NLS-URA3* *P_ATG18_::hphNT1-P_7tet.1_-ATG18 fab1^S202A/S203A/S204A/T206A/S208A/S210A^* | This study |  |
|  |  |  |  |
|  |  |  |  |
|  |  |  |  |
| yRL1290 | [BY4741] MATa; *his3∆1 leu2∆0 met15∆0 mNeonGreen-TOR1 ura3∆0::P_RNR2_-tetR-NLS-tup1-P_7tet.1_-tetR-NLS-URA3 P_ATG18_::hphNT1-P_7tet.1_-ATG18 fab1^S202D/S203D/S204D/T206D/S208D/S210D^* | This study |  |
| yRL1390 | [yRL795] *IVY1-mCherry-V5::kanMX* | This study |  |
| yRL1392 | [yRL1170] *IVY1-mCherry-V5::kanMX* | This study |  |
| yRL1341 | [RKH94] *atg21∆::kanMX* | This study |  |
| yRL1360 | [RKH94] *hsv2∆::kanMX* | This study |  |
| yRL1343 | [RKH158] *atg21∆::kanMX* | This study |  |
| yRL1364 | [RKH158] *hsv2∆::kanMX* | This study |  |
| yRL1362 | [RKH94] *vps26∆::kanMX* | This study |  |
| yRL1366 | [RKH158] *vps26∆::kanMX* | This study |  |
| yRL860 | [C-SWAT GFP library] *VPS29-mNeonGreen::hphNT1* | (Meurer et al., 2018) |  |
| yRL1387 | [yRL795] *VPS35-mCherry-V5::kanMX* | This study |  |
| yRL1466 | [yRL910] *VPS26-mCherry-V5::kanMX* | This study |  |
| yRL1430 | [yRL795] *ura3∆0::P_RNR2_-tetR-NLS-tup1-P_7tet.1_-tetR-NLS-URA3 P_ATG18_::hphNT1-P_7tet.1_-mCherry-ATG18* | This study |  |
| yRL1311 | [N’ SWAT GFP] *URA3::P_NOP1_-GFP-AVT1* | (Yofe et al., 2016) |  |
| yRL1312 | [N’ SWAT GFP] *URA3::P_NOP1_-GFP-AVT3* | (Yofe et al., 2016) |  |
| yRL765 | [N’ SWAT GFP] *URA3::P_NOP1_-GFP-AVT6* | (Yofe et al., 2016) |  |
| yRL1314 | [N’ SWAT GFP] *URA3::P_NOP1_-GFP-VBA1* | (Yofe et al., 2016) |  |
| yRL1316 | [N’ SWAT GFP] *URA3::P_NOP1_-GFP-VBA4* | (Yofe et al., 2016) |  |
| yRL1407 | [C’ SWAT GFP] *YPQ1-mNeonGreen::hphNT2* | (Meurer et al., 2018) |  |
| yRL976 | [C’ SWAT GFP] *YPQ2-mNeonGreen::hphNT2* | (Meurer et al., 2018) |  |
| yRL828 | [C’ GFP] *PMC1-GFP::HIS3* | (Huh et al., 2003) |  |
| yRL1347 | [N’ SWAT GFP] *URA3::P_NOP1_-GFP-VNX1* | (Yofe et al., 2016) |  |
| yRL1349 | [N’ SWAT GFP] *URA3::P_NOP1_-GFP-VHC1* | (Yofe et al., 2016) |  |
| yRL1350 | [N’ SWAT GFP] *URA3::P_NOP1_-GFP-MNR2* | (Yofe et al., 2016) |  |
| yRL1352 | [C’ GFP] *YBT1-GFP::HIS3* | (Huh et al., 2003) |  |
| yRL1354 | [C’ GFP] *BPT1-GFP::HIS3* | (Huh et al., 2003) |  |
| yRL1357 | [C’ SWAT GFP] *YVC1-mNeonGreen::hphNT2* | (Meurer et al., 2018) |  |
| yRL1358 | [C’ SWAT GFP] *YCF1-mNeonGreen::hphNT2* | (Meurer et al., 2018) |  |
| yRL1465 | [C’ GFP] *ATG27-GFP::HIS3* | (Huh et al., 2003) |  |
| yRL162 | [YL516] *DIP5-pHluorin::kanMX* | This study |  |
|  |  |  |  |
|  |  |  |  |
|  |  |  |  |
|  |  |  |  |
|  |  |  |  |
|  |  |  |  |
|  |  |  |  |
|  |  |  |  |
