# Supplementary material for: Vacuoles provide the source membrane for TORC1-containing signaling endosomes
Source: J Cell Biol. 2025 Mar 7;224(5):e202407021. doi: 10.1083/jcb.202407021 (PMC11893502; doi:10.1083/jcb.202407021)
Supplement: Table S2 — shows plasmids used in this study. [file jcb_202407021_tables2.docx]

**Table S2. Plasmids used in this study**

| **ID** | **Name** | **Description** | **Source** |
| --- | --- | --- | --- |
| pRL127 | pRS413 | CEN/ARS, *HIS3* | (Sikorski and Hieter, 1989) |
| pRL129 | pRS415 | CEN/ARS, *LEU2* | (Sikorski and Hieter, 1989) |
| pRL130 | pRS416 | CEN/ARS, *URA3* | (Sikorski and Hieter, 1989) |
| pRL479 | p1379 | CEN/ARS, *MET15* | (Hatakeyama et al., 2019) |
| pRL910 | pML104i/gTOR1-N | 2µ, *URA3*, *P_GAP_-SpCas9*, *P_SNR52_-gTOR1-N* | (Kira and Noda, 2021) |
| pRL886 | BYP9806 | *CSE4-mNeonGreen-HIS3* | (Okada et al., 2021) |
| pRL913 | pRL913 | *P_TOR1_-mNeonGreen-linker-TOR1^1-426^* | This study |
| pRL942 | FRP2365 | Integrative, *URA3, P_RNR2_-tetR-NLS-tup1-P_7tet.1_p-tetR-NLS* | (Azizoğlu et al., 2021) |
| pRL940 | FRP2350 | *hphNT1-P_7tet.1_-3xFLAG* | (Azizoğlu et al., 2021) |
| pRL1007 | pRS416-3FLAG-ATG18 | CEN/ARS, *URA3*, *3xFLAG-ATG18* | This study |
| pRL1022 | pRS416-3FLAG-ATG18T56E | CEN/ARS, *URA3*, *3xFLAG-ATG18^T56E^* | This study |
| pRL1025 | pRS416-3FLAG-ATG18SLoop | CEN/ARS, *URA3*, *3xFLAG-ATG18^Y367K/D369M/K373Y/M375D^* | This study |
| pRL1027 | pRS416-3FLAG-ATG18FGGG | CEN/ARS, *URA3*, *3xFLAG-ATG18^R285G/R286G^* | This study |
| pRL397 | pRS415-GFP-ATG8 | CEN/ARS, *LEU2*, *P_CUP1_-GFP-ATG8* | Christian Ungermann lab |
| pRL250 | pRS415-mCherry-GTR1 | CEN/ARS, *LEU2*, *mCherry-GTR1* | This study |
| pRL1034 | WTC_846_-mCherry | *hphNT1-P_7tet.1_-mCherry* | This study |
