# Supplementary figures and images for: Vacuoles provide the source membrane for TORC1-containing signaling endosomes
Source: J Cell Biol. 2025 Mar 7;224(5):e202407021. doi: 10.1083/jcb.202407021 (PMC11893502; doi:10.1083/jcb.202407021)

**F** *atg18Δ* mNG-Tor1 +3FLAG-Atg18 variant

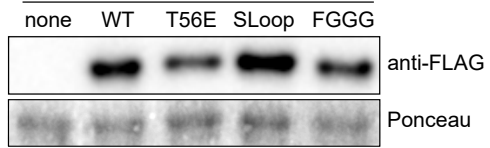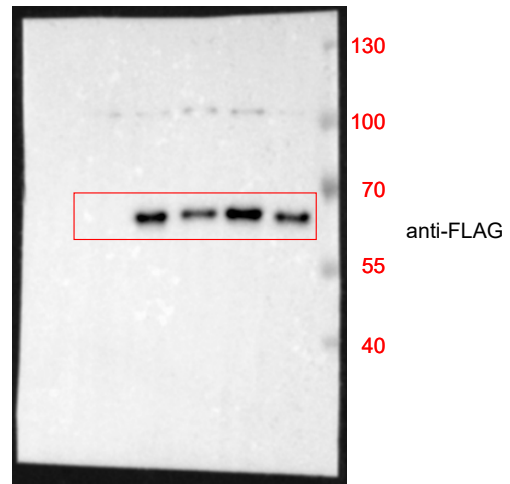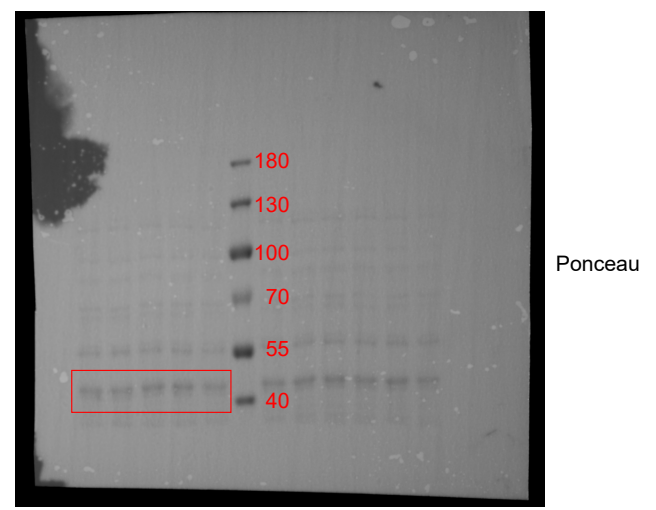

Supplement: SourceData F2 — is the source file for Fig. 2. [file jcb_202407021_sourcedataf2.pdf]

**A**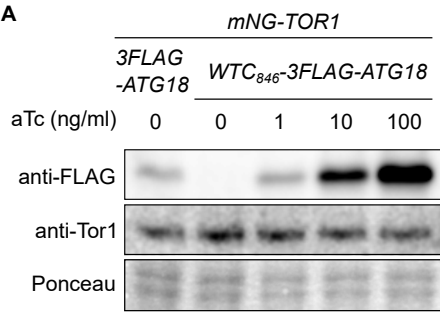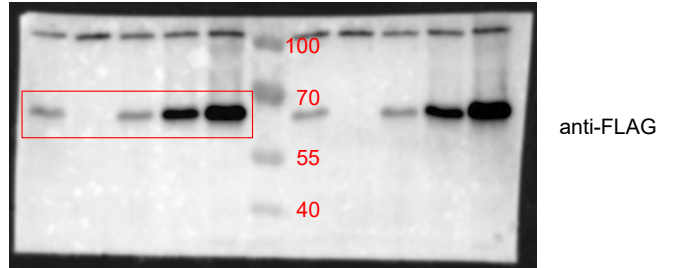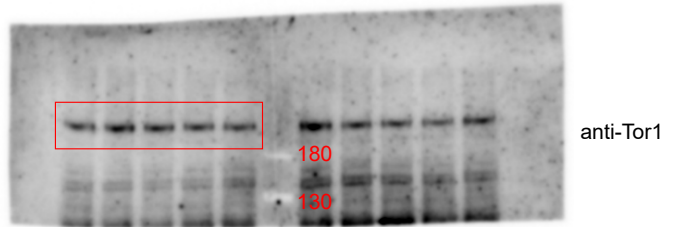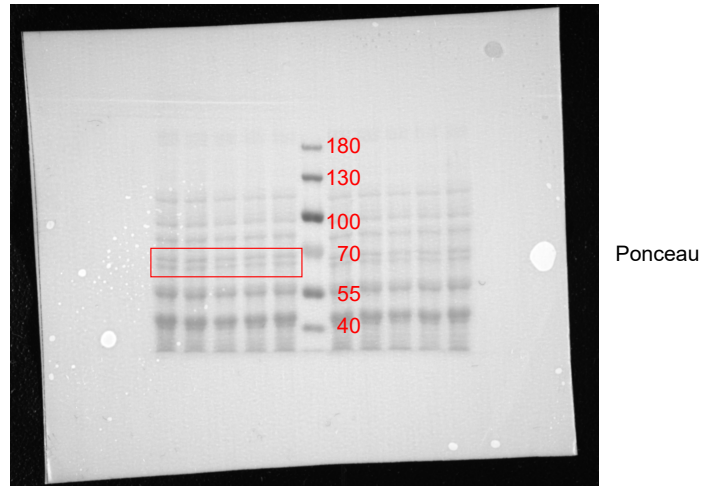

Supplement: SourceData F3 — is the source file for Fig. 3. [file jcb_202407021_sourcedataf3.pdf]

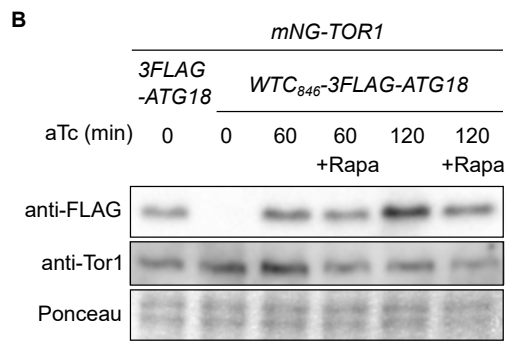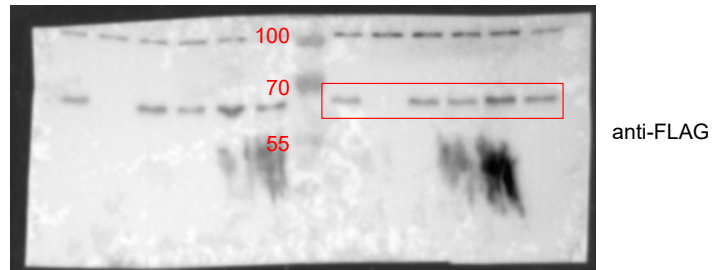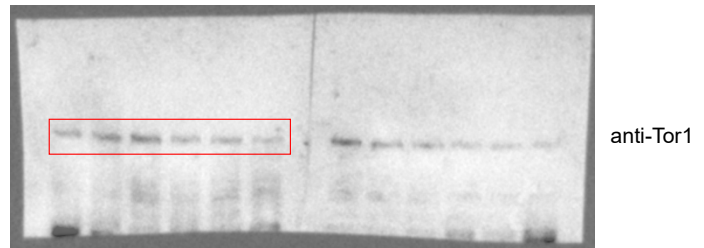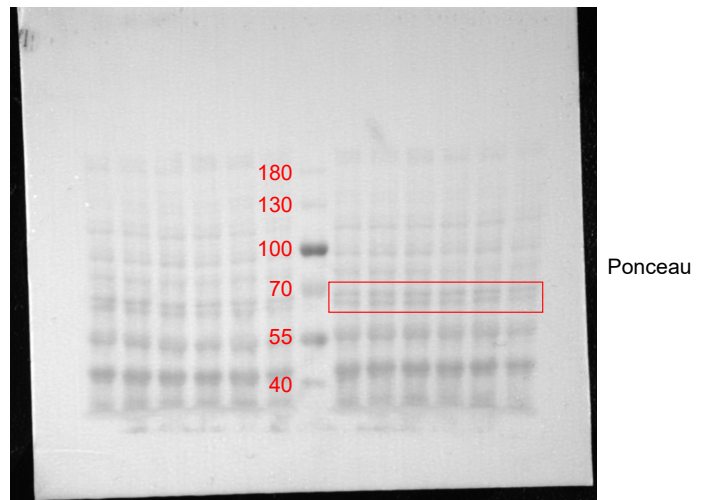

Supplement: SourceData F6 — is the source file for Fig. 6. [file jcb_202407021_sourcedataf6.pdf]

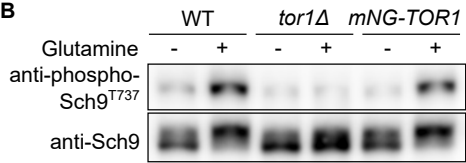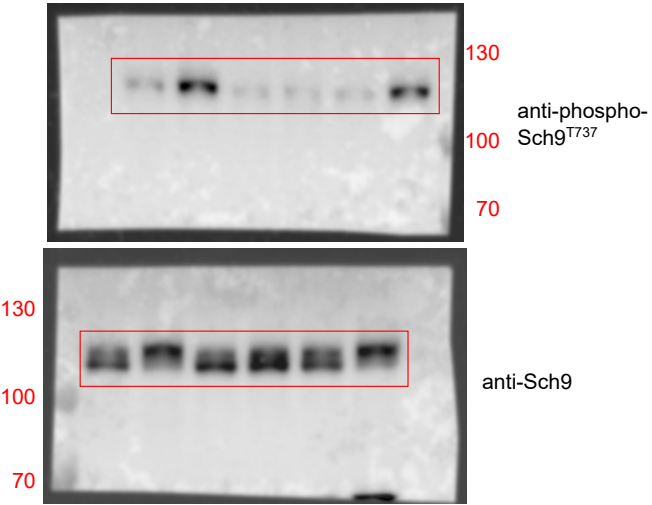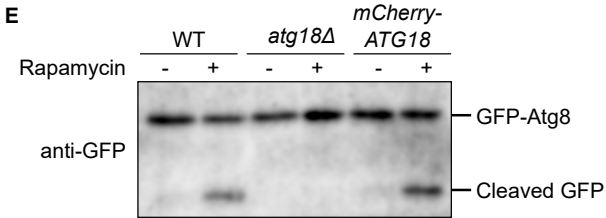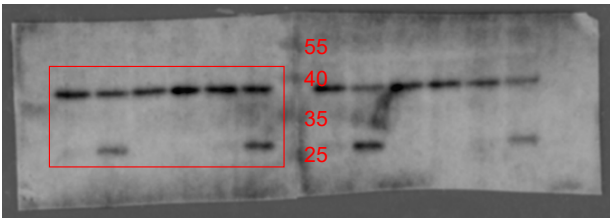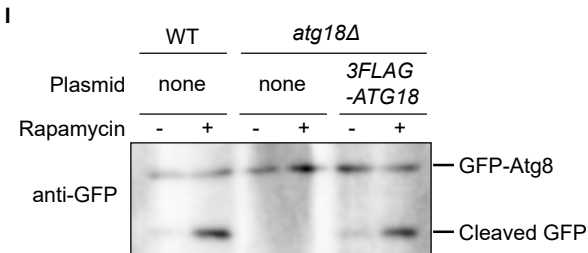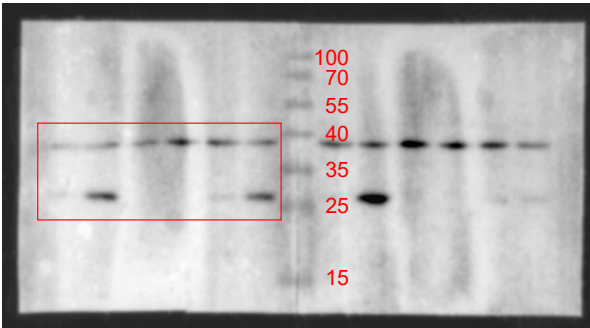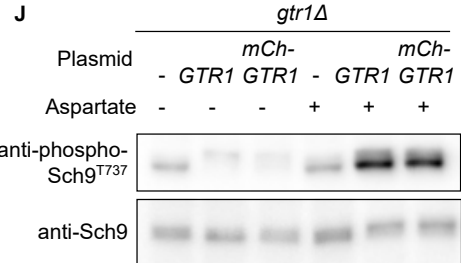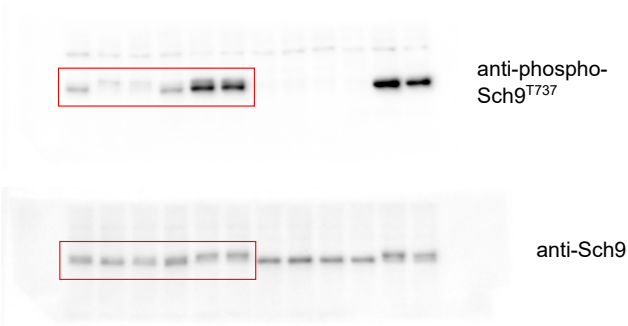

Supplement: SourceData FS1 — is the source file for Fig. S1. [file jcb_202407021_sourcedatafs1.pdf]

**A***WTC<sub>846</sub>-3FLAG-ATG18*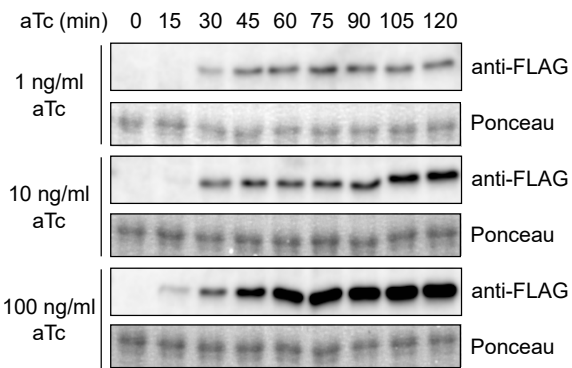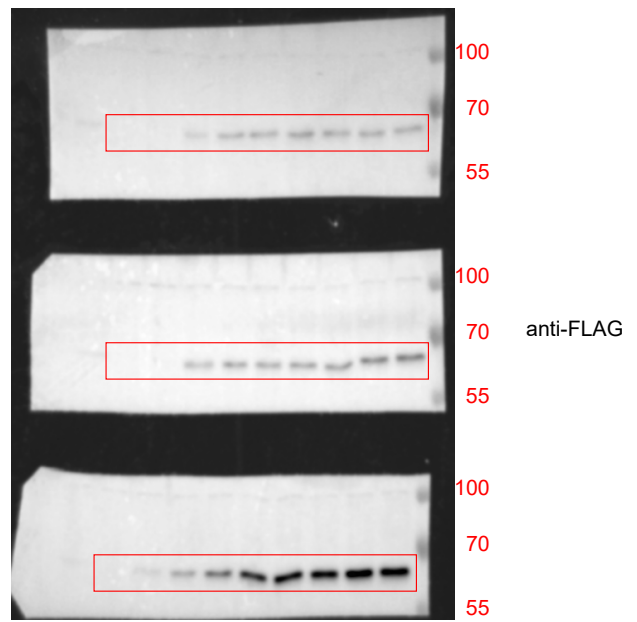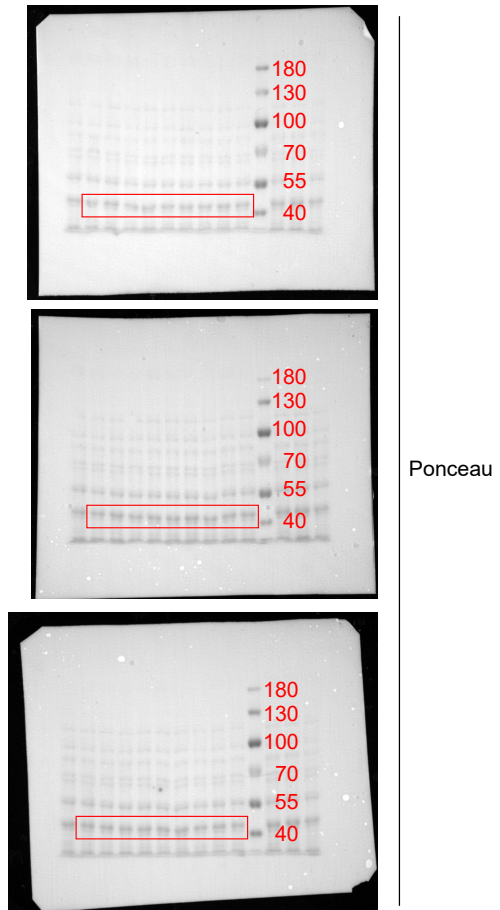

Supplement: SourceData FS4 — is the source file for Fig. S4. [file jcb_202407021_sourcedatafs4.pdf]
